# Assessing cerebral blood flow, oxygenation and cytochrome c oxidase stability in preterm infants during the first 3 days after birth

**DOI:** 10.1038/s41598-021-03830-7

**Published:** 2022-01-07

**Authors:** Ajay Rajaram, Daniel Milej, Marianne Suwalski, Lilian Kebaya, Matthew Kewin, Lawrence Yip, Sandrine de Ribaupierre, Victor Han, Mamadou Diop, Soume Bhattacharya, Keith St. Lawrence

**Affiliations:** 1 Imaging Program, Lawson Health Research Institute, London, ON Canada; 2 Department of Medical Biophysics, Western University, London, Canada; 3 Division of Neonatal-Perinatal Medicine, Department of Pediatrics, London Health Sciences Centre, London, ON N6A 3K7 Canada

**Keywords:** Developmental biology, Neuroscience, Biomarkers

## Abstract

A major concern with preterm birth is the risk of neurodevelopmental disability. Poor cerebral circulation leading to periods of hypoxia is believed to play a significant role in the etiology of preterm brain injury, with the first three days of life considered the period when the brain is most vulnerable. This study focused on monitoring cerebral perfusion and metabolism during the first 72 h after birth in preterm infants weighing less than 1500 g. Brain monitoring was performed by combining hyperspectral near-infrared spectroscopy to assess oxygen saturation and the oxidation state of cytochrome c oxidase (oxCCO), with diffuse correlation spectroscopy to monitor cerebral blood flow (CBF). In seven of eight patients, oxCCO remained independent of CBF, indicating adequate oxygen delivery despite any fluctuations in cerebral hemodynamics. In the remaining infant, a significant correlation between CBF and oxCCO was found during the monitoring periods on days 1 and 3. This infant also had the lowest baseline CBF, suggesting the impact of CBF instabilities on metabolism depends on the level of blood supply to the brain. In summary, this study demonstrated for the first time how continuous perfusion and metabolic monitoring can be achieved, opening the possibility to investigate if CBF/oxCCO monitoring could help identify preterm infants at risk of brain injury.

## Introduction

Premature birth is defined as a gestational age (GA) less than 37 weeks and has shown to strongly correlate with the development of adverse neurological outcomes such as cognitive and behavioural deficits and more severe disorders such as cerebral palsy^1–3^. The duration of gestation and an infant’s weight at birth are factors that influence the likelihood of adverse effects. One of the most common brain injuries associated with preterm birth is intraventricular hemorrhaging (IVH), which is characterized by bleeding in the germinal matrix and surrounding white matter. IVH has been found to occur in 20–25% of preterm infants born with very low birth weights (VLBW, < 1500 g) and typically occurs within the first 72 h after birth^4^. Diagnosis is performed using cranial ultrasound (cUS) to visualize and grade cerebral hemorrhages. In most centers, standard practice calls for imaging within the first week of life and again within the first month^5^. A drawback with cUS is that it is not a prognostic technique as it only detects damage that has already occurred.

Although the pathogenesis of IVH is multifactorial, unstable cerebral blood flow (CBF), leading to periods of ischemia, is considered a contributing factor due to the confluence of a number of factors^6^: an underdeveloped cerebrovascular system^7^, the absence of adequate cerebral autoregulation (i.e., the ability to maintain constant CBF during changes in blood pressure)^8^, and little tolerance to flow reductions given the already very low basal CBF^9^. Studies have shown the potential of near-infrared spectroscopy (NIRS) monitoring of tissue saturation (StO_2_) as a means of detecting precursors of preterm brain injury^10–12^. Correlating StO_2_ to arterial blood pressure has demonstrated that a sizable fraction of preterm infants has impaired cerebral autoregulation^8^. The SafeBoosC Phase II randomized clinical trial found that maintaining StO_2_ above 55% reduced the burden of hypoxia^13,14^. However, a link between StO_2_ and improved neurodevelopmental outcome has yet to be established^15^, likely due to inconsistencies in StO_2_ recordings between oximeters and the fact that StO_2_ is affected by multiple factors, including CBF, blood oxygen content, and tissue metabolism^16,17^.

Cerebral blood flow can be monitored directly using the flow-sensitive derivative of NIRS, diffuse correlation spectroscopy (DCS)^18^. In turn, the blood flow index (BFi) obtained from DCS can be combined with StO_2_ to measure an index of the cerebral metabolic rate of oxygen^19–25^. NIRS offers an alternative means of directly assessing metabolism by measuring changes in the oxidation state of cytochrome c oxidase (oxCCO), the terminal enzyme in the mitochondrial electron transport chain. Due to its low tissue concentration relative to hemoglobin, hyperspectral (hs) NIRS is optimal for monitoring oxCCO to avoid crosstalk between chromophores^26^. Combining hsNIRS with DCS offers the ability to evaluate the impact of fluctuations in CBF on cellular oxygen metabolism. Considering that cerebral energy requirements can be maintained during reductions in CBF by a compensatory increase in oxygen extraction^27–29^, detecting concurrent reductions in CBF and oxCCO could be of greater clinical significance—indication of possible hypoxia—than changes in perfusion or oxygenation alone.

With the goal of providing continuous bedside monitoring of CBF and oxCCO, a hybrid hsNIRS/DCS device was built (NNeMo: Neonatal NeuroMonitor)^30^. The aim of this study was to evaluate CBF and oxCCO stability within the first 72 h of life in preterm infants less than 32 weeks GA and weighing less than 1500 g. It was hypothesized that oxCCO would remain relatively independent of CBF fluctuations due to compensatory changes in oxygen extraction. As a corollary, a stronger temporal correlation between CBF and StO_2_ was expected, considering the sizable hemodynamic contributions to StO_2_.

## Results

Optical data were acquired from nine preterm infants on the first and third days of life. Between 80 and 90% of parents consented to participate in this study. The first monitoring period began on average at 5.9 ± 6.2 h after birth (range: 1 to 18 h) and the second at 53.8 ± 8.2 h (range: 44.5 to 61 h). Data from the first patient were excluded due to excessive ambient light that resulted in substantial signal artifacts. For subsequent acquisitions, the optical probes were covered at the site of contact using a thin blanket to minimize signal from ambient light sources. Data from the remaining eight infants are reported in this study. Average clinical parameters are provided in Table 1 and individual patient values can be found in the supplementary material (Table S1). On average, 233 ± 68 min (range: 145 to 340 min) of data were analyzed on day one and 254 ± 36 min (range: 210 to 290 min) on day three. One infant was diagnosed with IVH on the first day: a bilateral bleed with Grade II in the right hemisphere and Grade I on the left. This infant was the only one given inotropic support. All patients survived the neonatal period.

**Table 1:** Patient demographics and clinical metrics.

Gestational age at birth (weeks + days)	28 ± 2 (24 + 5 to 31 + 1)
Birth weight (g)	1123 ± 344
Sex (n)	5 Male; 3 Female
Apgar score 5 min	6.7 ± 3.2 (1 to 9)
Apgar score 10 min	8.3 ± 1.0 (7 to 9)
Mode of delivery	Vaginal (3), Caesarian (5)
Doses of antenatal steroids	1 dose (1), 2 doses (7)
Delayed cord clamping	Yes (5), No (0)
Mode of ventilation	6: continuous positive airway pressure, 2: invasive mechanical ventilation
Use of inotrope	Yes (1); No (7)
IVH diagnosis (n, grade)	1, Grade II right/Grade I left
Left ventricular output (LVO, ml/kg/min)	214 ± 185, day 1; 185 ± 68, day 3
Ejection fraction (EF, %)	62.7 ± 8.1, day 1; 64.3 ± 5.8, day 3
Presence of patent ductus arteriosus	7/7, day 1; 4/7, day 3
Arterial Blood Pressure (ABP, systolic/diastolic) (mmHg)	55/34 ± 13/11, day 1; 57/31 ± 12/5, day 3
Heart Rate (HR, beats per min)	161 ± 11, day 1; 156 ± 8, day 3
Respiratory Rate (RR, breaths per min)	59 ± 11, day 1; 50 ± 17, day 3
Arterial oxygen saturation (SaO_2_, %)	92.3 ± 7.3, day 1; 95.5 ± 2.6, day 3

Table 1 also includes cardiac measurements from targeted neonatal echocardiography (TnECHO). Note, TnECHO results are from the first seven patients, as imaging data were not recorded from the last infant due to an archiving error with the echo machine. Two patients had low left ventricular output (i.e. < 150 ml/kg/min) on both days, and one patient had a slightly low ejection fraction (EF = 51%) on day 1. No statistically significant change was found between days 1 and 3 for any of the parameters listed in Table 1.

**Figure 1:**
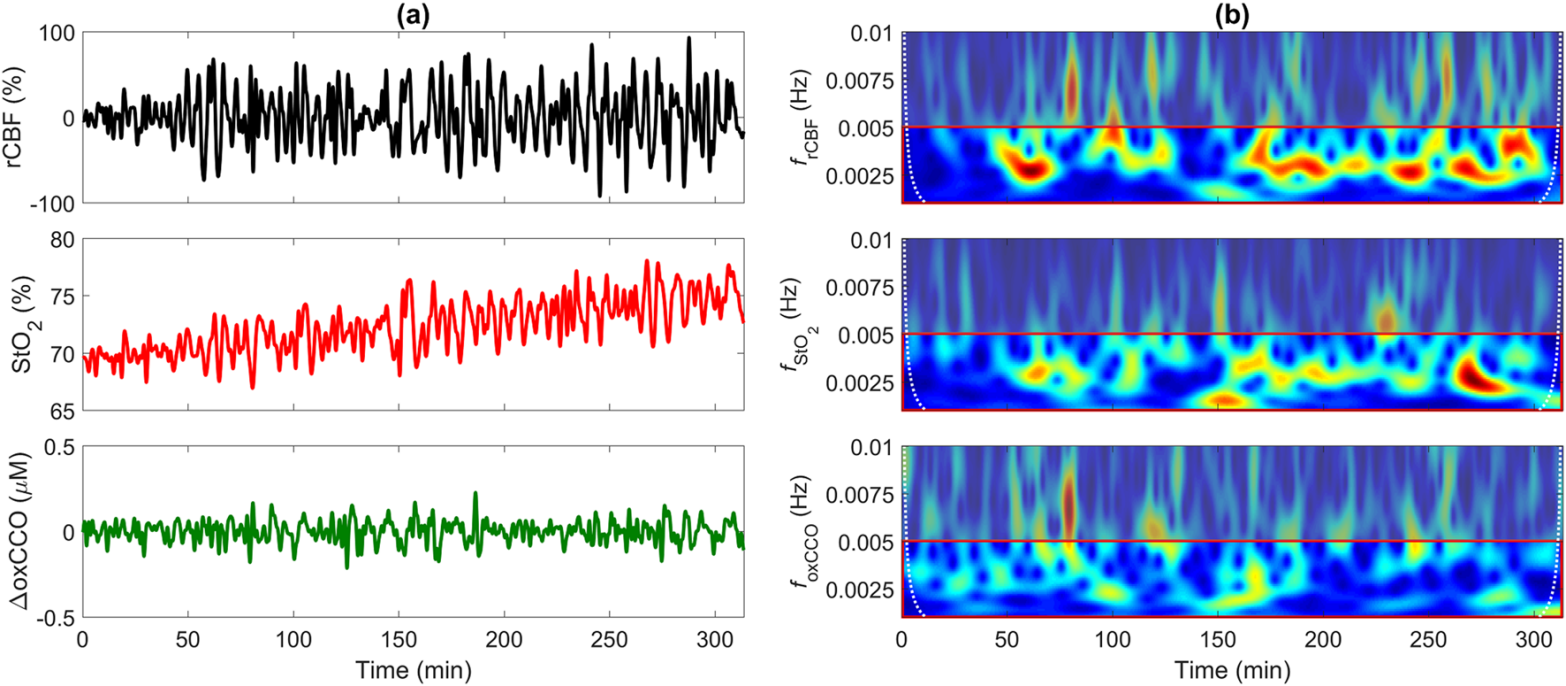
(**a**) Time courses of relative cerebral blood flow (rCBF), tissue saturation (StO_2_), and change in the oxidation state of cytochrome c oxidase (ΔoxCCO) for one patient on the first day of life. (**b**) Corresponding frequency-time plots generated by wavelet transform of rCBF, StO_2_, and ΔoxCCO. The red box highlights the frequency band of notable CBF oscillation (0.001 to 0.005 Hz). The dotted white lines indicate the cone of influence. Wavelet values outside this region were considered distorted due to edge artefacts^56^.

Average baseline BF_i_ was 8.8 ± 3.6 × 10^–9^ cm^2^/s on day 1 (range: 4.7 to 16.6 × 10^–9^ cm^2^/s) and 16.8 ± 7.7 × 10^–9^ cm^2^/s on day 3 (range: 11.1 to 33.0 × 10^–9^ cm^2^/s). There was a significant increase in CBF between the two days (*p* = 0.022). Average baseline StO_2_ was 73.3 ± 11.9% on day 1 (range: 54.5 to 85.5%) and 80.1 ± 7.0% on day 3 (range: 71.9 to 94.5%), with no significant difference. Time courses of StO_2_, relative CBF (rCBF) and change in oxCCO (ΔoxCCO) on the first day of life for one infant are displayed in Fig. 1. This figure also includes the corresponding wavelet transforms for the three signals. These frequency-time heat maps illustrate the pronounced CBF oscillations in a narrow band of very low frequencies (0.0025–0.005 Hz) that were fairly consistent across the 5-h period. The standard deviation (SD) of rCBF across the monitoring period was calculated as a means of characterizing the magnitude of the blood flow oscillations, and in this case, SD = 32%. These oscillations were also evident in the corresponding StO_2_ time series but not in ΔoxCCO. For this patient (see supplementary Table S1 for their clinical characteristics), the hemodynamic oscillations were considerably dampened on the third day (SD = 5.7%; data not shown). A general trend of reduced fluctuations in CBF across participants between the two days was observed (SD = 13 ± 11% on day 1 versus 8 ± 7% on day 3); however, this trend was not statistically significant.

**Figure 2:**
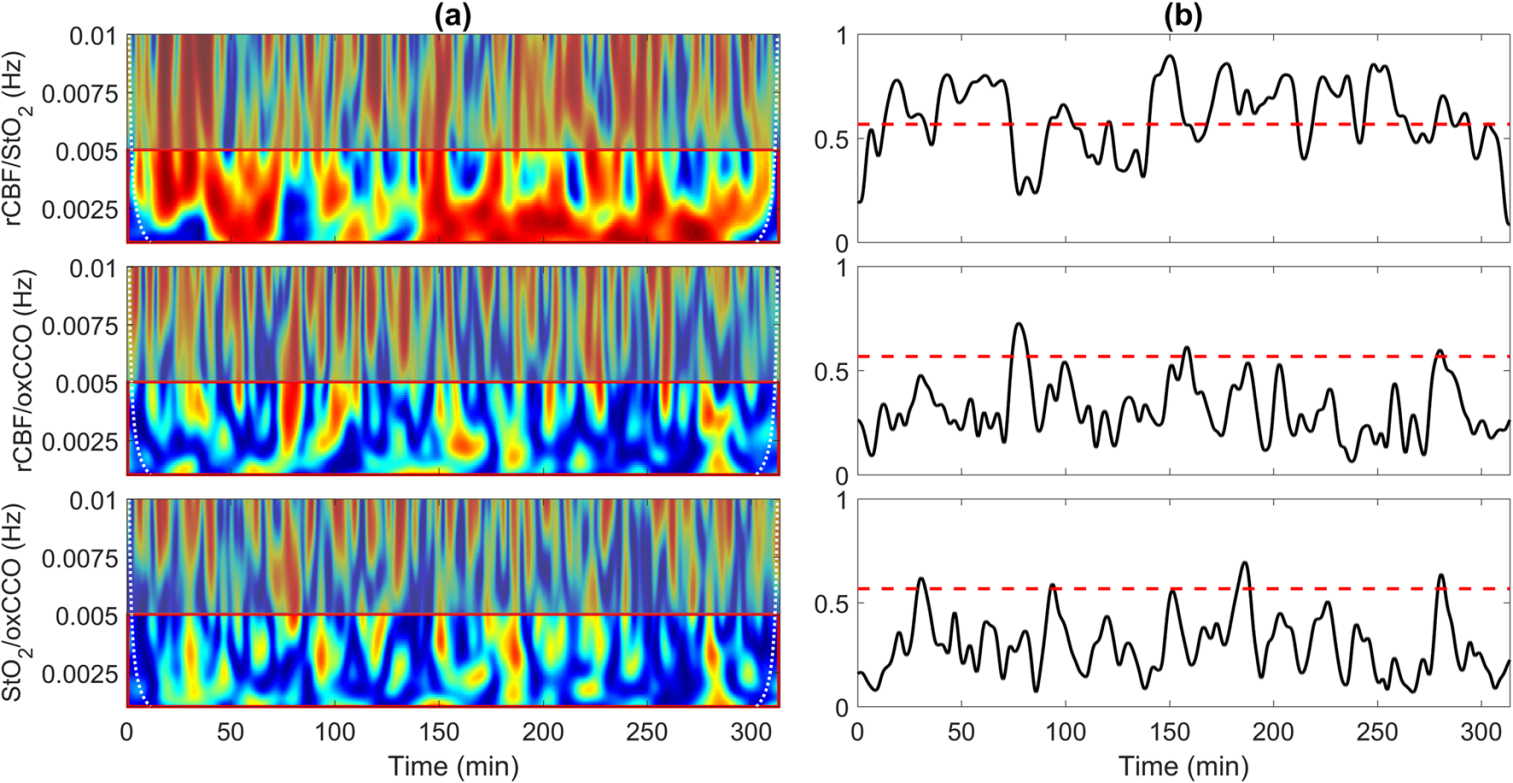
(**a**) Coherence between relative cerebral blood flow (rCBF) and tissue saturation (StO_2_), rCBF and oxidation state of cytochrome c oxidase (oxCCO), and StO_2_ and oxCCO for one patient on the first day of life. (**b**) Average coherence value in the frequency range 0.001 − 0.005 Hz [indicated by the red box in column (**a**)]. The red dashed line indicates the statistical threshold.

Figure 2a displays wavelet coherence of rCBF/StO_2_, rCBF/ΔoxCCO and StO_2_/ΔoxCCO for the same patient presented in Fig. 1. Average coherence from 0.001 to 0.005 Hz is shown in Fig. 2b. For this patient, the coherence between rCBF and StO_2_ was above the statistical threshold of 0.57 for 59% of monitoring period. The corresponding semblance plot (supplementary Fig. S1) indicated that rCBF and StO_2_ were predominately in-phase, with a mean semblance of 0.56 ± 0.23, which was greater than for all other patients (inter-subject mean = 0.27 ± 0.18). In contrast, the two coherence plots involving ΔoxCCO only reached significance for 4.5% and 4.8% of the monitoring period for rCBF/ΔoxCCO and StO_2_/ΔoxCCO, respectively. Similarly, mean semblance values for this patient were 0.02 ± 0.34 for rCBF/ΔoxCCO and 0.05 ± 0.39 for StO_2_/ΔoxCCO. For comparison, inter-subject means were -0.01 ± 0.1 and -0.01 ± 0.09 for rCBF/ΔoxCCO and StO_2_/ΔoxCCO, respectively.

**Figure 3:**
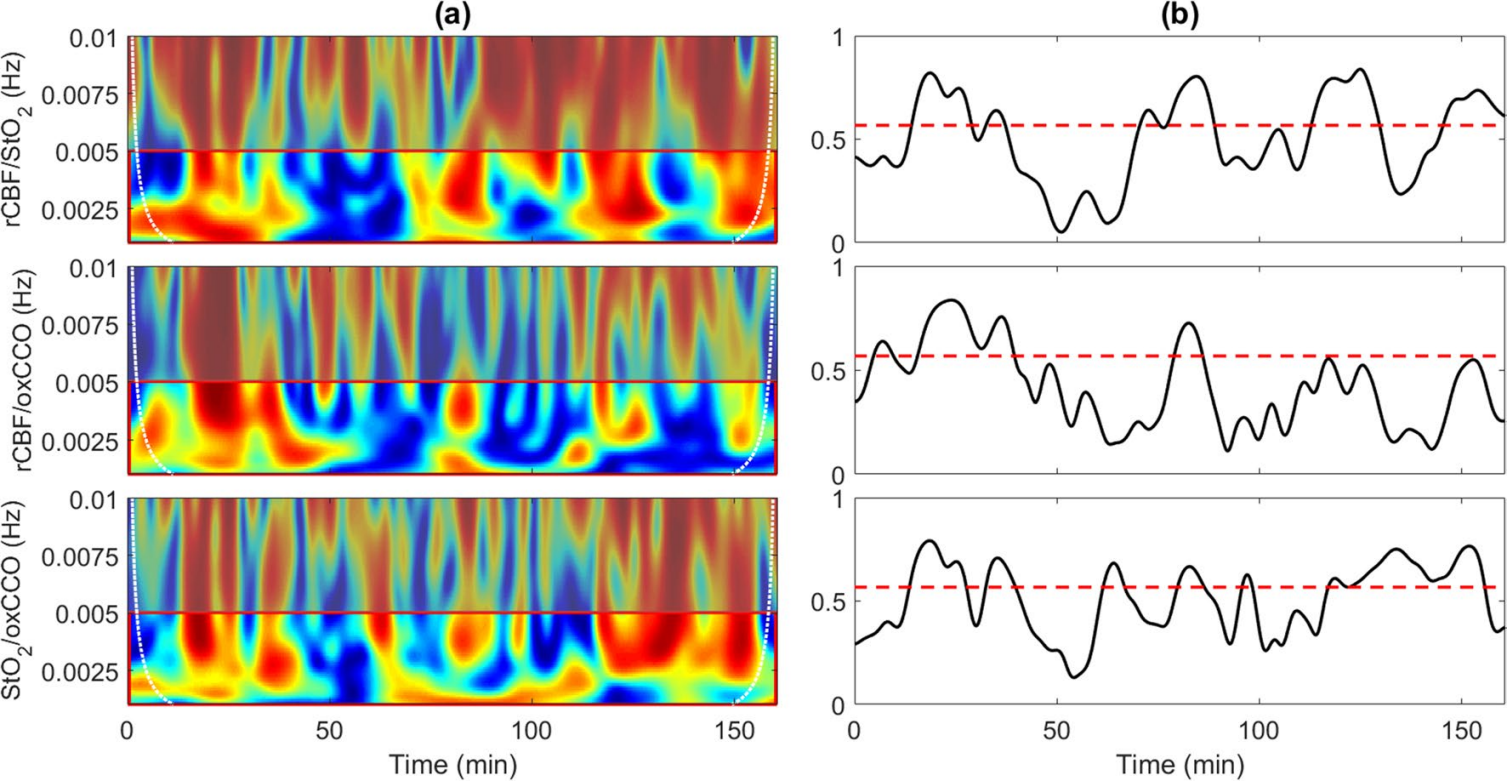
(**a**) Coherence between rCBF and StO_2_, rCBF and oxCCO, and StO_2_ and oxCCO for one patient on the first day of life. (**b**) Average coherence value in the frequency range 0.001−0.005 Hz. The red dashed line indicates the statistical threshold.

Figure 3 presents wavelet coherence for another patient who exhibited the greatest coherence with respect to CCO. In this case, data are presented from the monitoring period on day 1. Coherence was above the statistical threshold for 43% of the time for rCBF/StO_2_, 22% for rCBF/ΔoxCCO and 46% for StO_2_/ΔoxCCO. Similar patterns were also observed on day 3; that is, significance coherence was found for 32%, 17% and 44% of the monitoring period for rCBF/StO_2_, rCBF/ΔoxCCO and StO_2_/ΔoxCCO, respectively.

**Figure 4:**
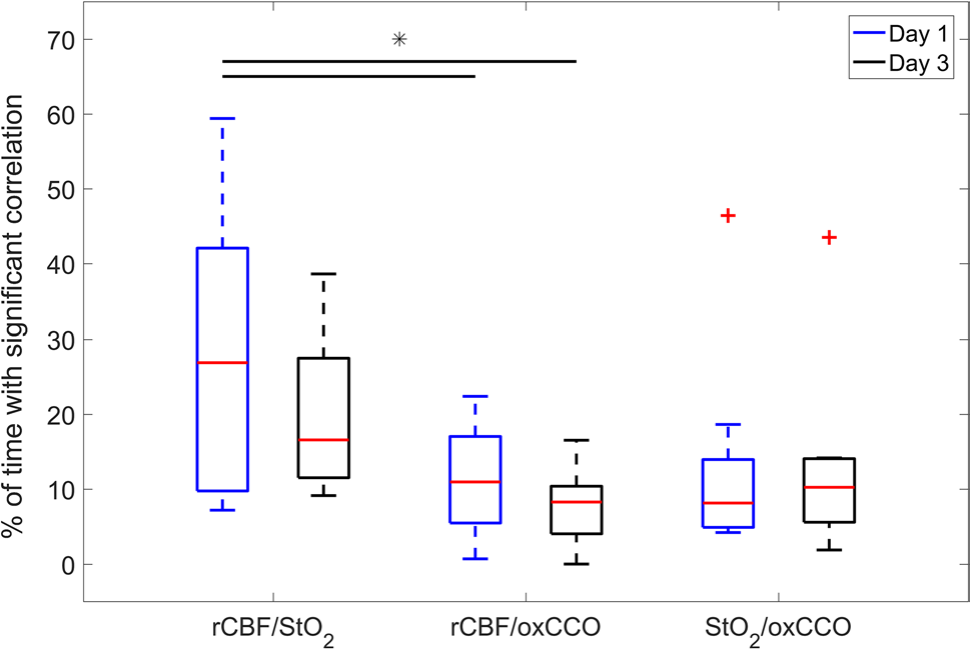
Boxplots of the fraction of time that coherence between two optical parameters reached the statistical threshold of 0.57. Data are presented separately for the two monitoring periods and for the three combinations of coherence estimates: rCBF/StO_2_, rCBF/ΔoxCCO and StO_2_/ΔoxCCO. Statistical outliers are indicated by + , and the bars indicate significant differences in coherence duration between rCBF/StO_2_ on day 1 and rCBF/ΔoxCCO on days 1 (*p* = 0.025) and 3 (*p* = 0.004). Details of each boxplot are as follows: rCBF/StO_2_ (day 1): median: 26.9, 75th percentile: 42.1, 25th percentile: 9.8; rCBF/StO_2_ (day 3): median: 16.6, 75th percentile: 27.5, 25th percentile: 11.5; rCBF/oxCCO (day 1): median: 11.0, 75th percentile: 17.0, 25th percentile: 5.5; rCBF/oxCCO (day 3): median: 8.3, 75th percentile: 10.4, 25th percentile: 4.0; StO_2_/oxCCO (day 1): median: 8.2; 75th percentile: 14.0; 25-th percentile: 4.9; and StO_2_/oxCCO (day 3): median: 10.3, 75th percentile: 14.1, 25th percentile: 5.6.

Figure 4 provides boxplots of the fraction of time that coherence estimates for rCBF/StO_2_, rCBF/ΔoxCCO and StO_2_/ΔoxCCO were greater than the statistical threshold determined from Monte Carlo simulations. Coherence was calculated for the frequency band from 0.001 to 0.005 Hz. A two-way ANOVA revealed that the duration of rCBF/StO_2_ coherence on day 1 was significantly greater that the corresponding coherence durations for rCBF/ΔoxCCO on both days. The two outliers identified in the StO_2_/ΔoxCCO coherence boxplots are from the patient presented in Fig. 3. If these outliers were excluded, then the rCBF/StO_2_ coherence duration on day 1 was also significantly greater that the values for StO_2_/ΔoxCCO on days 1 and 3.

## Discussion

This proof-of-concept study demonstrated the feasibility of concurrently monitoring cerebral perfusion and metabolism in VLBW preterm infants during the first 72 h of life. Continuous monitoring was achieved using a hybrid system combining hsNIRS and DCS. While hsNIRS is less common that conventional NIRS system that emit light at a few wavelengths, it has the advantage of providing a direct assessment of oxygen metabolism by measuring oxCCO, and there is an increasing interest in this metabolic marker in neonatal studies^31^. The challenge with combining hsNIRS and DCS is avoiding cross contamination, in particular, blocking the DCS laser that can easily saturate the spectrometer used to measure ΔoxCCO^28^. NNeMo avoids crosstalk by incorporating a multiplexing system to collect data from the two subsystems sequentially^30^. The trade-off with this approach is lower temporal resolution compared to hybrid designs that provide simultaneous CBF and StO_2_ monitoring using NIRS techniques involving only a few wavelengths^21,32,33^. However, the 7-s duration used to collect DCS and hsNIRS data was sufficient for monitoring very low frequency hemodynamic and metabolic fluctuations (i.e. those less than 0.01 Hz).

The primary outcome of the study was to confirm the hypothesis that cerebral metabolism remained relatively independent of cerebral blood flow in the majority (7/8) of patients, indicating that adequate oxygen delivery was maintained despite fluctuations in cerebral hemodynamics. Evidence is provided by the low coherence between ΔoxCCO and both rCBF and StO_2_, as shown in Fig. 4. The most dramatic illustration of the independence of oxCCO from CBF is the data set presented in Figs. 1 and 2. This patient exhibited large and sustained oscillations in rCBF throughout most of monitoring period on the first day. They had the lowest left ventricular output and tachypnea on day 1 (P5 in Table S1), which could contribute to flow instabilities given that impaired cerebral autoregulation is common in preterm infants^8^. Independent of the possible cause, this study demonstrated that the corresponding oxCCO time course exhibited only small variations (less than 0.1 μM) despite large blood flow fluctuations. To put the magnitude of these changes in context, reductions in oxCCO of the order of 1–1.5 µM were reported during desaturation events in term infants with hypoxic-ischemic encephalopathy^34^. The implication of stable oxCCO during variable CBF indicates that this patient was not experiencing cycles of cerebral hypoxia, which has been postulated to contribute to IVH^6^. In contrast to oxCCO, Fig. 2 illustrates that StO_2_ was strongly affected by fluctuations in CBF, as indicated by the significant rCBF/StO_2_ coherence throughout the five hours. This was confirmed by the rCBF/StO_2_ semblance plot (Fig. S1) that showed strong positive correlations between rCBF and StO_2_. A positive correlation is expected when there is no change in metabolism since increases in CBF will cause a greater oxygenation of venous blood and, vice versa, reduced CBF will lower venous oxygenation.

Of the eight patients, only one exhibited hemodynamic/metabolic coherence that was comparable in duration to the coherence between rCBF and StO_2_ (Fig. 3; P5 in Table S1). Furthermore, the time series of average coherence for rCBF/StO_2_, rCBF/ΔoxCCO and StO_2_/ΔoxCCO followed similar patterns with good agreement in terms of the specific times of significance coherence. Interestingly, this infant had the lowest baseline BFi, which was 47% lower than the group average. This finding suggests that cerebral metabolism is less tolerant to hemodynamic instabilities in patients with low baseline CBF, presumably because at low flow the brain is close to the lower threshold of sufficient oxygen delivery. More studies are required to confirm this postulate considering this patient did not develop IVH. In all likelihood, any link between flow/metabolic coherence and the development of brain injury shortly after birth will depend on the duration of coherence and the magnitude of CBF changes. It would also be useful in future studies to acquire continuous recordings of ABP and SaO_2_ to investigate which clinical features are likely driving the rCBF/ΔoxCCO coherence. Nevertheless, these preliminary results highlight the value of directly monitoring CBF with DCS, as opposed to relying on StO_2_ as a surrogate marker. It should be noted that although DCS does not measure CBF in classic units of perfusion, BFi has been shown to closely track CBF in both neonatal animal models and human infants^24,35–38^.

Average StO_2_ values (73.3 ± 11.9% on day 1 and 80.1 ± 7.0% on day 3) were comparable to values reported by Roche-Labarbe et al. (between 75 to 83%) for preterm infants less than a GA < 30 weeks and Noori et al. (mean of 79%) for infants less than 27 weeks of age^20,39^. However, a larger study reported average StO_2_ closer to 65% during the first 72 h of life for preterm infants^40^. Discrepancies are likely related to differences in NIRS devices (hsNIRS in the current study, frequency-domain NIRS in the Roche-Labarbe study, and continuous-wave systems in the other two studies), source-detector distances, and patient populations. Similar to previous studies^39,40^, we found no significant difference in StO_2_ between the two monitoring periods that were 6 and 54 h after birth, respectively. In contrast, a significant increase in CBF of 45% was found between the two monitoring periods. To the best of our knowledge, this is the first study to monitor cerebral perfusion directly during the first 72 h of life in preterm infants. The mean baseline BFi on day 3 (16.8 ± 7.7 × 10^–9^ cm^2^/s) was in good agreement with a previous DCS study involving preterm infants^41^, but lower than the value reported for healthy term infants (27.1 ± 15.6 × 10^–9^ cm^2^/s)^42^. This difference could be due to brain maturation, although BFi estimates will also be affected by the chosen ${\mu }_{a}$ and ${\mu }_{s}^{\prime}$ values.

As a feasibility study, the sample size was too small to assess the relationship between flow/metabolism coupling and the occurrence of IVH. In fact, only one patient was diagnosed with Grade 1 IVH, which was detected by cUS at the beginning of the monitoring period on day 1 (P3 in Table S1). This patient exhibited similar stability metrics compared to the rest of the patients with a relative duration of significant rCBF/StO_2_ coherence of 41% and a corresponding rCBF/oxCCO coherence of 12%. Despite the limited sample size, the study demonstrated that monitoring CBF, StO_2_ and oxCCO could begin shortly after birth and continue for extended periods. A monitoring period of 6 h was selected for this initial study; however, the amount of usable data was reduced to 233 ± 68 min on the first day and 254 ± 36 min on day three due to signal artefacts related primarily to patient handling. No evidence of skin irritation caused by the probe holder was found in any of the sessions, indicating that continuously monitoring throughout the first 72 h is achievable.

An alternative to measuring oxCCO is to calculate the cerebral metabolic rate of oxygen from CBF, SaO_2_ and StO_2_ measurements, which has the advantage that StO_2_ can be measured by more common NIRS technologies than hsNIRS^19–25^. Despite measuring CBF and StO_2_ in the current study, a comparison between these two metabolic markers was not conducted as the required SaO_2_ data could not be retrieved from the clinical monitors. This study limitation highlights a potential advantage to measuring oxCCO since it is a direct marker of metabolism that does not require other input parameters. Clinical interest in oxCCO monitoring will likely increase given the feasibility of using low-cost spectrometers^43,44^.

In summary, this is the first study to demonstrate how continuous cerebral perfusion and metabolic monitoring in VLBW preterm infants can be performed by combining hsNIRS and DCS. The application of wavelet coherence analysis demonstrated that the temporal correlation between StO_2_ and rCBF on the first day of life was significantly greater than the corresponding correlation between ΔoxCCO and either rCBF or StO_2_. This finding indicates that cerebral oxygen metabolism is generally independent of hemodynamic fluctuations. However, significant coherence between ΔoxCCO and rCBF was found in the patient with the lowest baseline blood flow index, suggesting that the combination of low cerebral blood flow and hemodynamic instability can affect metabolism. Further studies are required to determine if CBF/oxCCO monitoring could help identify preterm infants at greater risk of IVH.

## Methods

### Patient population

This study was approved by the Western University Health Sciences Research Ethics Board, which adheres to the guidelines of the Canadian Tri-Council Policy Statement: Ethical Conduct for Research Involving Humans in accordance with the Declaration of Helsinki. Patient recruitment was conducted in the neonatal intensive care unit at the Children’s Hospital, London Health Sciences Center. Informed -parental consent was obtained for all patients recruited for this study. Participants were infants born less than 32 weeks GA and weighing less than 1500 g. Neuromonitoring consisted of two periods, each up to 6 h in duration, that were within the first 72 h of life. The first period started as soon as clinically feasible following birth and resuscitation, and the second period started at 48 h post-natal age. In each session, cUS was performed to diagnose cerebral hemorrhaging, which was graded according to the Papile scale^45^ and targeted neonatal echocardiography (TnECHO) used to assess cardiac output (i.e. left ventricular output and ejection fraction) and to screen for patent ductus arteriosus^46^. Heart rate (HR), respiratory rate (RR) and arterial oxygen saturation (SaO_2_) were recorded from the clinical monitor at the beginning and end of each period. Note, the monitors used in the unit did not provide the option to save the continuous recordings of these parameters. Arterial blood pressure (ABP) was measured non-invasively at the beginning of each period.

### Study design

**Figure 5:**
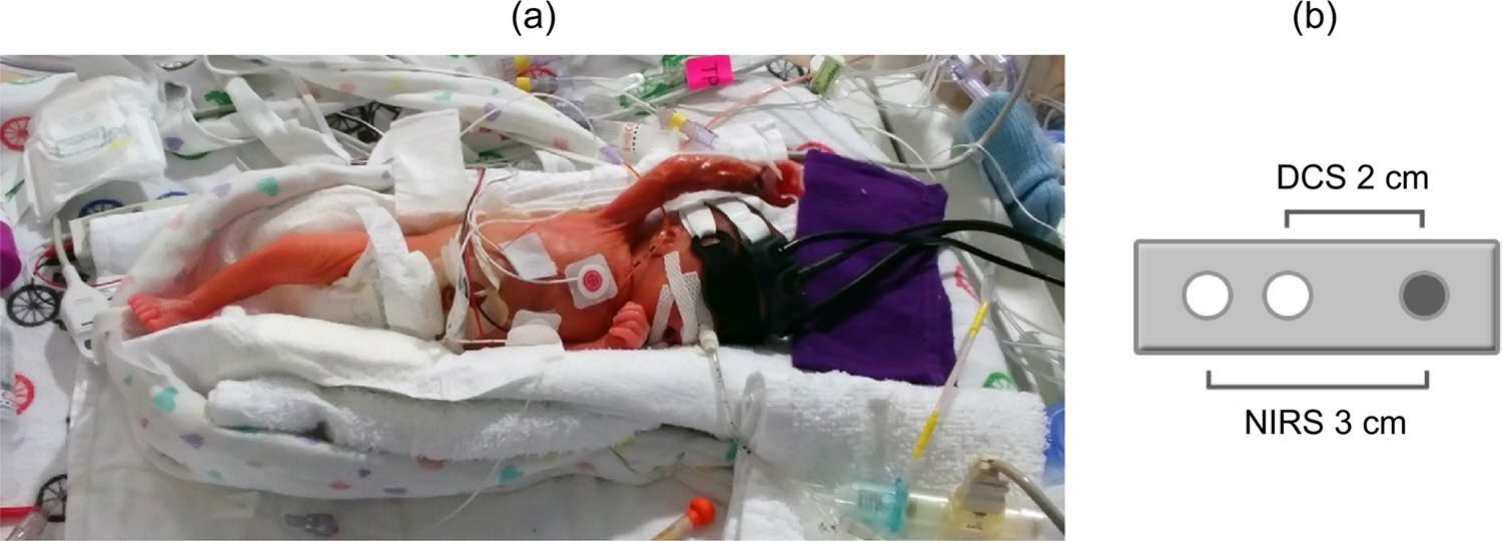
(**a**) Premature infant with optical probes secured to the forehead and a phototherapy eye shield, (**b**) schematic of probe holder showing the position of the NIRS (3-cm SDD) and DCS (2-cm SDD) sources and the common detection location (shaded circle). Probe holder was 5 × 2 × 1 cm.

Once transferred to the NICU from the birthing suite and following initial vitals by nursing staff, the optical probes were secured to the scalp above the frontoparietal cortex using a 3-D printed probe holder and adjustable strap (Fig. 5A). The optical fibers were bent 90° at the point of contact on the head for ease of use, and the optical prober holder was designed to be light, flexible and non-abrasive (Fig. 5B; dimension of 5 × 2 × 1 cm; Flexible Resin, Form 2, Formlabs, Somerville, MA, USA). The design of the probe holder enabled it to be used with infants requiring ventilation with continuous positive airway pressure (CPAP) as it could be positioned under the CPAP cap and tube. The infant’s eyes were shielded using phototherapy eye goggles as a precaution. Two optical fiber bundles (hsNIRS and DCS sources) directed light to the head, while a third (common detection) collected diffusely reflected light. The source-detector distance was 3 cm for hsNIRS and 2 cm for DCS. A smaller distance was chosen for DCS as it inherently has greater sensitivity to the brain^47^. Power levels for the two light sources and spot size incident on the scalp were adjusted to meet ANSI standards for skin exposure. Continuous DCS and hsNIRS data sets were saved in one-hour intervals throughout each monitoring period. One member of the research team remained at the bedside during data acquisition to log any events or clinical procedures that could affect the quality of the optical signals. Following each monitoring period, the probe holder was removed, and the skin was assessed for redness or irritation. This workflow was repeated for the monitoring session on the third day.

### Instrumentation

The NNeMo system combines hsNIRS and DCS by incorporates a multiplexing shuttering system to cycle between acquisitions from the two subsystems^29,30^. For this study, hsNIRS and DCS data were each acquired at a sampling frequency of 4 Hz in consecutive 3-s intervals, resulting in full data sets every 7 s (with a 0.5-s dead-time between subsystems). The hsNIRS system employed a 20-W halogen bulb filtered outside of 600 to 1000 nm (Hl-2000-HP, Ocean Insight, Delay Beach, FL, USA) and a spectrometer (iDus 420, Andor, Oxford Instruments, Abingdon, UK; 548–1085 nm bandwidth; 1.65 nm resolution; P&P Optica, Waterloo, ON, Canada). An optical fiber bundle (2.4 mm outer diameter, core = 30 µM, numerical aperture (NA) = 0.55), Loptek, Berlin, Germany) directed light towards the scalp, while a second set of fibers (three linearly aligned fibers; diameter = 2 mm, core = 30 µm, NA = 0.55, Loptek) collected diffusely reflected light. The DCS system consisted of a long-coherence laser (DL785-100-S, CrystaLaser, Reno, NV, USA) and a four-channel single-photon counting module (SPCM-AQR-15-FC, Excelitas Technologies, Montreal, QC, Canada). The laser was coupled to four fibers (core = 200 µm, NA = 0.22, Loptek), and four single-mode fibers were used for detection (core = 8 µm, NA = 0.12, Loptek). The output for the SPCM was fed into a PCIe-6612 data acquisition board that generated intensity autocorrelation functions using in-house developed software: LabVIEW 2017 SP1 (National Instruments, https://www.ni.com/en-ca/support/downloads/software products/download.labview.html#306,351) and MATLAB 2016b (MathWorks, https://www.mathworks.com/help/releases/R2016b/index.html^48^.

### Data analysis

#### Quantifying StO_2_ and changes in oxCCO by hsNIRS

Each monitoring period began by collecting a dark spectrum (dark_λ_) acquired with the emission source turned off. A reference spectrum (reference_λ_) was collected to characterize the spectral properties intrinsic to the instrument. Each reflectance spectrum, R(λ), was determined by:^49^

1 $$R\left( \lambda \right) = \frac{{{\text{spectrum}}_{\lambda } - {\text{dark}}_{\lambda } }}{{{\text{reference}}_{\lambda } - {\text{dark}}_{\lambda } }}$$

where spectrum_λ_ refers to intensity measurements as a function of wavelength λ.

A derivative spectroscopy approach was applied to R(λ) to quantify the tissue water fraction (WF) and baseline concentrations of oxy- and deoxy-hemoglobin ($Hb{O}_{2}^{b}$ and $H{b}^{b}$, respectively)^24,50^. The approach involves fitting the first and second derivatives of R(λ) with the solution to the diffusion approximation for a semi-infinite homogeneous medium. Light absorption and scattering parameters were input into the model solution as follows:

2 $$\mu_{a} \left( \lambda \right) = WF \cdot \varepsilon_{{H_{2} O}} \left( \lambda \right) + Hb^{b} \cdot \varepsilon_{Hb} \left( \lambda \right) + HbO_{2}^{b} \cdot \varepsilon_{{HbO_{2} }} \left( \lambda \right)$$

3 $$\mu_{s} ^{\prime}\left( \lambda \right) = A \cdot \left( {\frac{\lambda }{{800 {\text{nm}}}}} \right)^{ - \alpha }$$

where $\mu_{a} \left( \lambda \right)$ is the absorption coefficient, $\varepsilon$ refers to the molar extinction coefficient of each chromophore, $\mu_{s} ^{\prime}\left( \lambda \right)$ is the reduced scattering coefficient, *α* is the scattering power, and A is the value of $\mu_{s} ^{\prime}\left( \lambda \right)$ at λ = 800 nm.

Following baseline analysis, a modified Beer-Lambert Law approach based on the UCLn algorithm was utilized to determine time-varying changes in the concentrations of HbO_2_, Hb and oxCCO^26^:

4 $$\left[ {\begin{array}{*{20}c} {\Delta HbO_{2} \left( t \right)} \\ {\Delta Hb\left( t \right)} \\ {\Delta oxCCO\left( t \right)} \\ \end{array} } \right] = \frac{1}{{DP}}\left[ {\begin{array}{*{20}c} {\varepsilon _{{HbO_{2} }} \left( {\lambda _{1} } \right)} & {\varepsilon _{{Hb}} \left( {\lambda _{1} } \right)} & {\varepsilon _{{oxCCO}} \left( {\lambda _{1} } \right)} \\ \vdots & \vdots & \vdots \\ {\varepsilon _{{HbO_{2} }} \left( {\lambda _{n} } \right)} & {\varepsilon _{{Hb}} \left( {\lambda _{n} } \right)} & {\varepsilon _{{oxCCO}} \left( {\lambda _{1} } \right)} \\ \end{array} } \right] \cdot \left[ {\begin{array}{*{20}c} {\Delta A\left( {\lambda _{1} ,t} \right)} \\ \vdots \\ {\Delta A\left( {\lambda _{n} ,t} \right)} \\ \end{array} } \right]$$

where Δ denotes a change relative to baseline concentration, DP is the differential pathlength (set to 4.9 based on previous literature^51^), and A is the measured change in attenuation. StO_2_ as a function of time was determined by:

5 $$StO_{2} = \frac{{HbO_{2}^{b} + {\Delta }HbO_{2} \left( t \right)}}{{HbO_{2}^{b} + {\Delta }HbO_{2} \left( t \right) + Hb^{b} + {\Delta }Hb\left( t \right)}}$$

#### Monitoring CBF by DCS

To determine BF_i_, each normalized intensity autocorrelation curve was converted to electric field autocorrelation data using the Siegert relation^18^:

6 $$g_{2} \left( {\rho ,\tau } \right) = 1 + \beta \frac{{\left| {G_{1} \left( {\rho ,\tau } \right)} \right|^{2} }}{{I\left( {\rho ,\tau } \right)^{2} }}$$

where g_2_(ρ, τ) represents the measured normalized intensity autocorrelation as a function of source-detector distance (*ρ*) and correlation time (τ), G_1_(ρ, τ) is the electric field autocorrelation function, < *I*(ρ, τ) > is the average intensity, and β is the coherence factor. G_1_ was fit with the solution to the diffusion approximation for a semi-infinite homogenous medium based on the assumption of pseudo-Brownian motion of light scatterers^52,53^. The fitting was performed by incorporating changes in ${\mu }_{a}\left(\lambda \right)$ determined by hsNIRS and assuming ${\mu }_{s}^{\prime}\left(\lambda \right)$ = 8 cm^-1^. The average BF_i_ for the first 15 min of data acquisition was used to define the baseline ${\overline{BF} }_{i}$. All BF_i_ values were normalized to ${\overline{BF} }_{i}$ to generate a time series of fractional changes in CBF: $rCBF=\left({BF}_{i}-{\overline{BF} }_{i}\right)/{\overline{BF} }_{i}$.

### Data processing and statistical analysis

The first step was to remove data sections that exhibited substantial signal artifacts. The criteria were based on large signal variations in either a hemoglobin time course or the corresponding blood flow index that exceeded four times the temporal standard deviation and persisted for at least 15 min. A comparison to the clinical log was conducted to establish the most likely cause of each artifact (see supplementary Fig. S2 as an example). The most common cause, which occurred at least once in every monitoring period, was patient handling related to checking vital signs, patient repositioning, and responding to desaturation events (i.e., a decrease in SaO_2_ below 85%). In addition, six periods were disrupted by clinical procedures including cUS and x-ray imaging, phototherapy and surfactant treatment. Data corresponding to any identified artifact were subsequently removed from all time courses. The second step was to filter the StO_2_, rCBF, and ΔoxCCO time courses using an inverse wavelet transform with a Morlet wavelet (the MATLAB function *cwt*)^54–56^ to remove frequencies greater than 0.01 Hz and less than 0.001 Hz. The final step was to correct each time course for motion artifacts using an algorithm that utilizes a moving standard deviation and spline interpolation^57^.

Absolute baseline measurements of BF_i_ and StO_2_ were determined for each monitoring period by averaging across the first 15 min of data acquisition. A paired t-test was used to compare baseline BF_i_ and StO_2_ values from the two periods, as well as all other physiological parameter measured on days 1 and 3. All statistical analysis was conducted using a statistical toolbox (MATLAB R2020b) and significance was defined as *p* < 0.05. All data are presented as mean ± standard deviation.

Wavelet coherence, which has been used in a number of neonatal brain monitoring studies^23,58–60^, was used to assess the temporal correlation between the three dynamic signals across the frequency range 0.001 to 0.005 Hz: rCBF and StO_2_, rCBF and ΔoxCCO, and StO_2_ and ΔoxCCO. Coherence ranges from 0 to 1 and reflects the cross-correlation between two time series as a function of frequency, with a value of 0 indicating no correlation and 1 indicating complete agreement. Statistical significance coherence values were determined from Monte Carlo simulations of 1000 data pairs of simulated red noise. Coherence was computed for each pair, and the statistical threshold for the experimental data was based on values greater than 95% of the simulated values^56^. The final step was to calculate the duration of significance coherence for rCBF/StO_2_, rCBF/ΔoxCCO and StO_2_/ΔoxCCO for each patient and monitoring session. A two-way analysis of variance (ANOVA) was used to investigate differences in coherence durations for rCBF/StO_2_, rCBF/ΔoxCCO and StO_2_/ΔoxCCO from the two sessions. Boxplots were used to display the results with statistical outliers determined as points greater than q3 + w × (q3 − q1) or less than q1 − w × (q3 − q1) where q is the quartile number and w is the whisker length.

To assess the phasic relationship between the signals, wavelet semblance was calculated^55^. Semblance is the instantaneous phase difference and ranges from − 1 for signals completely out of phase to 1 for signals in-phase.

## Supplementary Information

Supplementary Information.
