# Supplementary material for: Assessing cerebral blood flow, oxygenation and cytochrome c oxidase stability in preterm infants during the first 3 days after birth
Source: Sci Rep. 2022 Jan 7;12:181. doi: 10.1038/s41598-021-03830-7 (PMC8741949; doi:10.1038/s41598-021-03830-7)
Supplement: Supplementary file 1 — Supplementary Information. [file 41598_2021_3830_MOESM1_ESM.pdf]

Table S1: Individual patient clinical metrics acquired on day 1

Metric	P1	P2	P3†	P4	P5‡	P6	P7	P8¶
GA (weeks)	30	29	24	26	29	25	28	31
Weight (kg)	1.57	1.40	0.67	0.63	1.42	1.01	1.15	1.14
Sex	M	M	M	M	M	F	F	F
Mode of Ventilation£	1	1	2	1	1	1	2	1
APGAR 5 min	9	1	3	9	7	9	4	9
APGAR 10 min	9	7	7	9	9	9	7	9
LVO (ml/kg/min)	120	508	172	284	70	182	160	-
EF (%)	51	68	58	61	58	75	68	-
ABP(mm Hg)	68/39	47/25	34/24	66/47	60/37	47/24	75/52	51/19
HR (bpm)	160	-	163	177	166	161	169	147
RR (bpm)	50	-	-	38	80	53	48	63
SaO_2 (%)	98	-	94	73	99	92	94	93
$Time_M$ (hr)	5.5	4	18	2.5	13	1	2	1

GA: Gestational age, LVO: Left ventricular output, EF: Ejection fraction, ABP: Arterial blood pressure, HR: Heart rate, RR: Respiratory rate, SaO_2: arterial saturation, $Time_M$: Time between birth and the beginning of optical neuromonitoring.

† Grade 2 (right) intraventricular hemorrhage diagnosed by cranial ultrasound prior to optical monitoring

‡ Patient with pronounced cerebral blood flow (CBF) oscillations on day 1.

¶ Patient with significant correlation between CBF and the oxidation state of cytochrome c oxidase (oxCCO).

£ Mode of ventilation: 1 = Non-invasive, 2 = Invasive

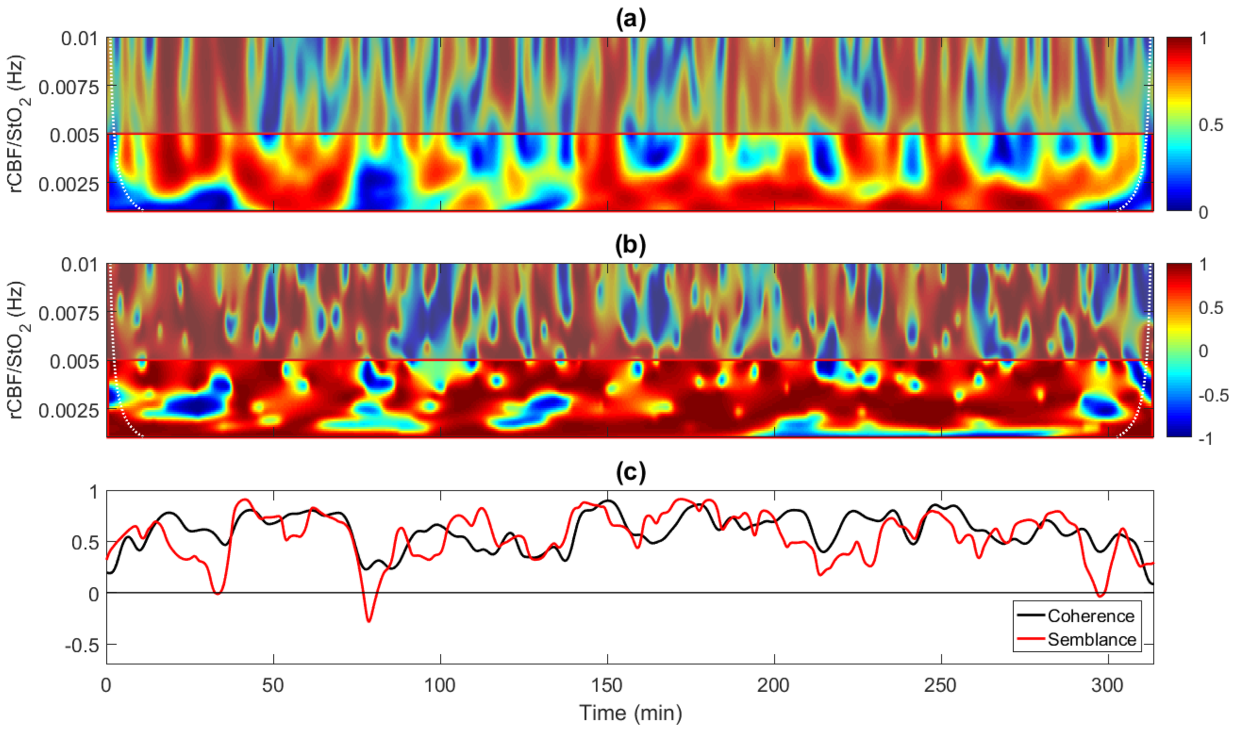

Figure S1. (A) Coherence between relative cerebral blood flow (rCBF) and tissue saturation (StO_2) for the same patient shown in Figure 2. (B) Corresponding semblance plot. Average value was 0.56 ± 0.23 in the frequency range 0.001 - 0.005 Hz (indicated by the red box). (C) Coherence (black line) and semblance (red line) time courses averaged over the frequency range 0.001 - 0.005 Hz.

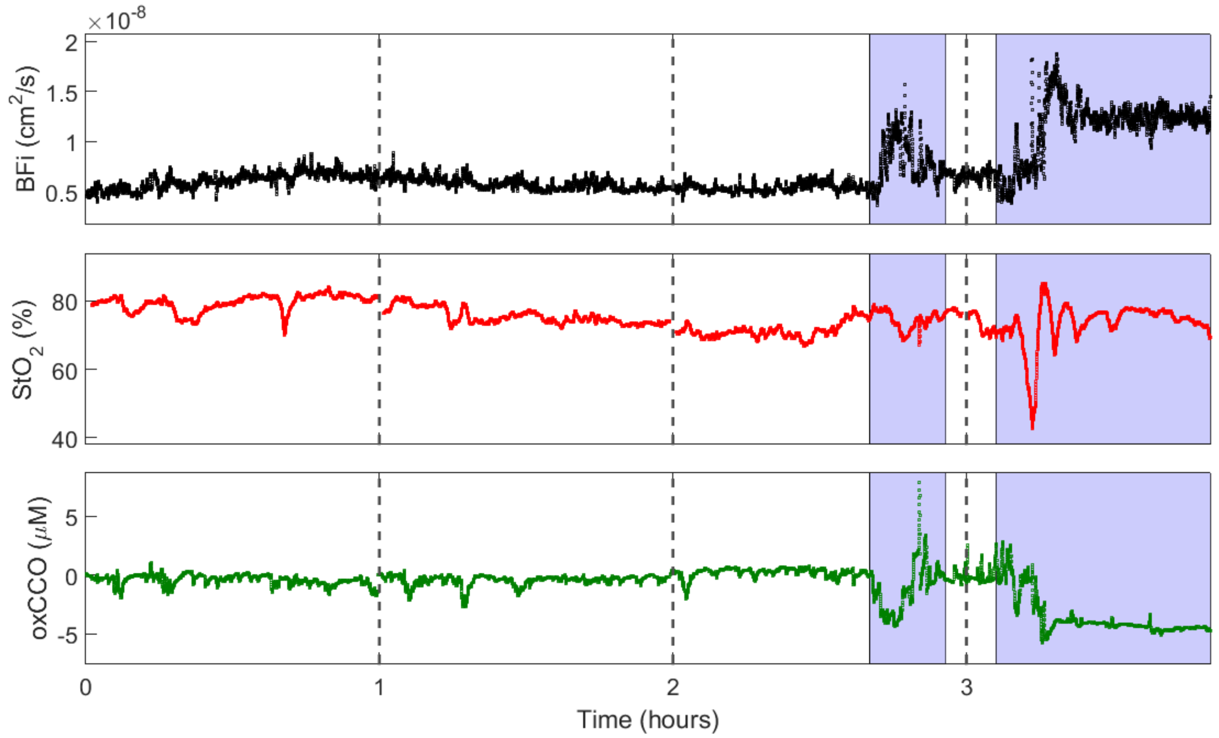

Figure S2. Time courses of the blood flow index (BFi), tissue saturation (StO_2), and change in the oxidation state of cytochrome c oxidase (ΔoxCCO) from one patient. Vertical dashed lines indicate one-hour blocks of data that were saved individually. Shaded blue areas represents time periods identified as containing artefacts, which were subsequently removed.
